# *In* *Vivo* Detection of Intracellular Signaling Pathways in Developing Thymocytes

**DOI:** 10.1155/2000/97820

**Published:** 2000

**Authors:** Alison M. Michie, Juan Carlos Zúñiga-Pflücker

**Affiliations:** Department of Immunology 1 King' College Circle University of Toronto Ontario Toronto M5S 1A8 Canada

**Keywords:** Cellular signaling, FTOC, T-cell development, thymus, transfection

## Abstract

Information regarding the intracellular signaling processes that occur during the development of T cells has largely been obtained with the use of transgenic mouse models, which although providing invaluable information are time consuming and costly. To this end, we have developed a novel system that facilitates the *In* *Vivo* analysis of signal transduction pathways during T-lymphocyte development. This approach uses reporter-plasmids for the detection of intracellular signals mediated by the mitogen-activated protein kinase or cyclic AMP-dependent protein kinase. Reporter-plasmids are transfected into thymocytes in fetal thymic organ culture by accelerated DNA/particle bombardment (gene gun), and the activation of a signaling pathway is determined in the form of a standard luciferase assay. Importantly, this powerful technique preserves the structural integrity of the thymus, and will provide an invaluable tool to study how thymocytes respond to normal environmental stimuli encountered during differentiation within the thymic milieu. Thus, this method allows for the monitoring of signals that occur in a biological time frame, such as during differentiation, and within the natural environment of differentiating cells.


*Developmental Immunology*, 2000, Vol. 8(1), pp. 31-45
Reprints available directly from the publisher
Photocopying permitted by license only

© 2000 OPA (Overseas Publishers Association)
N.V. Published by license under the
Harwood Academic Publishers imprint,
part of The Gordon and Breach Publishing Group.
Printed in Malaysia

### *In Vivo* Detection of Intracellular Signaling Pathways in Developing Thymocytes

ALISON M. MICHIE* and JUAN CARLOS ZÚÑIGA-PFLÜCKER

*Department of Immunology, 1 King's College Circle, University of Toronto, Toronto, Ontario M5S 1A8, Canada*

*(Received October 29, 1999)*

Information regarding the intracellular signaling processes that occur during the development of T cells has largely been obtained with the use of transgenic mouse models, which although providing invaluable information are time consuming and costly. To this end, we have developed a novel system that facilitates the *in vivo* analysis of signal transduction pathways during T-lymphocyte development. This approach uses reporter-plasmids for the detection of intracellular signals mediated by the mitogen-activated protein kinase or cyclic AMP-dependent protein kinase. Reporter-plasmids are transfected into thymocytes in fetal thymic organ culture by accelerated DNA/particle bombardment (gene gun), and the activation of a signaling pathway is determined in the form of a standard luciferase assay. Importantly, this powerful technique preserves the structural integrity of the thymus, and will provide an invaluable tool to study how thymocytes respond to normal environmental stimuli encountered during differentiation within the thymic milieu. Thus, this method allows for the monitoring of signals that occur in a biological time frame, such as during differentiation, and within the natural environment of differentiating cells.

*Keywords:* Cellular signaling, FTOC, T-cell development, thymus, transfection

*Abbreviations:*cyclic AMP, cyclic adenosine monophosphate, CREB, cyclic AMP response, element binding protein, CMV, cytomegalovirus, FTOC, fetal thymic organ culture, DN, $CD4^-$ $CD8^-$ double negative thymocytes, DP, $CD4^+$ $CD8^+$ double positive thymocytes, MAPK, mitogen-activated protein kinase, PKA, cyclic AMP dependent protein kinase, PMA, phorbol-12-myristate-13-acetate, SCF, stem cell factor, TCR, T cell antigen receptor

### INTRODUCTION

Thymocyte development is characterized by the stage-specific expression of CD4 and CD8 surface molecules (Godfrey and Zlotnik, 1993; Shortman and Wu, 1996; Zúñiga-Pflücker and Lenardo, 1996). The earliest thymic immigrants, arriving from the fetal liver or bone marrow, lack CD4 and CD8 expression ($CD4^-$ $CD8^-$, double negative (DN)) (Godfrey et al., 1993). Only thymocytes that successfully rearrange their TCR-β locus expand and differentiate to the $CD4^+$ $CD8^+$ (double positive, DP) stage; a checkpoint known as β-selection (Hoffman et al., 1996; von Boehmer and Fehling, 1997). Following β-selection, the initiation of TCR-α gene expression and rearrangement occurs (von Boehmer and Fehling, 1997). DP

* Corresponding author. Tel.: 416–978–8217, Fax: 416–978–1938, E-mail: alison.michie@utoronto.ca.

thymocytes expressing a complete αβ-TCR/CD3 complex undergo further checkpoints, positive and negative selection (Fowlkes and Schweighoffer, 1995; Kisielow and von Boehmer, 1990), which result in the generation of $CD4^+$ $CD8^-$ and $CD4^-$ $CD8^+$ mature thymocytes. The intracellular signaling pathways that surround these checkpoints and drive the maturation process of thymocytes are not well-defined. Transgenic mouse models have enabled the identification of key signaling components necessary during T-cell development, such as Ras, Rho GTPase, Lck, SLP-76, LAT, ZAP-70 (Clements et al., 1998; Galandrini et al., 1997; Levin et al., 1993; Molina et al., 1992; Negishi et al., 1995; Swan et al., 1995; Swat et al., 1996; Zhang et al., 1999). Although these studies are informative, they fail to provide a biological time frame for the activation of proximal and distal signaling pathways that occur at checkpoints during T cell development.

To study the regulation of gene expression or cellular signaling events during thymocyte development several experimental strategies are available. These approaches rely on the introduction of foreign DNA into thymocytes (Zúñiga-Pflücker et al., 1993). A wide variety of DNA transfection protocols have been developed, such as the use of retroviruses, liposomes, adenoviruses, gene gun-bombardment, and electroporation (Crompton et al., 1996; Michie and Zúñiga-Pflücker, 2000; Rooke et al., 1997; Sugawara et al., 1998; Suzuki et al., 1991; Zúñiga-Pflücker et al., 1993). Each of these approaches has its own merits and drawbacks, depending on the stage of development being studied and the type of assay system employed for analytical readout.

Retroviral infection of early fetal thymocytes allowed for stable expression of transduced genes during thymocyte differentiation (Crompton et al., 1996; Pallard et al., 1999; Sugawara et al., 1998). Crompton et al. (1996), achieved this by directly infecting fetal thymic lobes, and Sugawara et al. (1998) reconstituted fetal thymic lobes with thymocytes infected by coculture with a retroviral packaging cell line. Rooke et al. (1997) used adenovirus-mediated infection to transduce thymic stroma, as these quiescent cells are not susceptible to retroviral infection. Electroporation of thymocytes has also been carried out, and has the advantage of being easily applicable to thymocytes in cell suspension (Michie and Zúñiga-Pflücker, 2000; Nickoloff, 1995; Zúñiga-Pflücker et al., 1993). Thus, allowing for the isolation, by flow cytometry cell sorting or magnetic bead separation, of a particular target population of thymocytes to be transfected.

Previous innovations in tissue culture techniques resulted in the creation of an *in vitro* T-cell development system that is amenable to controlled experimentation; this technique is known as the fetal thymic organ culture (FTOC) (Anderson and Jenkinson, 1998; Anderson et al., 1996). FTOCs involve the culturing of thymic lobes in an air-liquid interphase, providing favorable conditions for normal growth and differentiation (Jenkinson et al., 1982). In contrast to thymocytes grown in standard suspension cultures, which normally undergo rapid apoptotic death, FTOCs maintain an intact thymic stromal architecture, thus providing an appropriate environment for thymocyte differentiation (Anderson and Jenkinson, 1998). Therefore, the FTOC system allows for the study of thymocyte development within its natural physiological *in vivo* setting.

We have developed an approach that uses an accelerated DNA/particle bombardment (gene gun) delivery system to transfect thymocytes in FTOC (Zúñiga-Pflücker et al., 1993). Here we have combined this non invasive means of DNA delivery into thymocytes with a novel reporter-plasmid system, which allows intracellular signaling cascades to be monitored via reporter-gene plasmids, such as luciferase and β-galactosidase. In this report, we have characterized the optimal conditions with which to study two signaling pathways within thymic lobes, involving mitogen-activated protein kinase (MAPK) and CREB-mediated signaling pathways through cyclic AMP-dependent protein kinase (PKA). The introduction of reporter-plasmids into thymic lobes without disrupting the thymic microenvironment permits the study of biochemical signaling events in real time and within a relevant biological setting.

### RESULTS

### Detection of Intracellular Signaling Pathways with Reporter-Plasmids

The cellular signaling processes that govern developmental checkpoints during T-cell maturation within the thymus are not well defined. Therefore, we have developed a technique that combines gene gun-mediated transfection of FTOCs with reporter-plasmids that allows intracellular signaling cascades to be monitored in an *in vivo* setting and in a biological time frame.

A novel system for the *in vivo* detection of different signal transduction pathways, such as those involving MAPK and PKA, involves the use of commercially available reporter-plasmids (Fig. 1). This approach takes advantage of three plasmids: the first one encodes for a fusion protein containing an activation domain derived from the specific substrate recognition site for each kinase (pFA-Elk or pFA2-CREB) and a DNA-binding protein domain derived from the yeast DNA transcriptional activator GAL4; the second plasmid encodes for the luciferase gene controlled by five repeats of a GAL4-binding element followed by a basic transcriptional promoter (TATATA) (pFR-Luc); the third plasmid provides a positive control, encoding for constitutively active version of the kinase to be tested (pFC-MEK1 or pFC-PKA), which is under the transcriptional control of strong enhancer/promoter elements. When the fusion-activator and luciferase-reporter plasmids are cotransfected into mammalian cells, activation-induced phosphorylation of the fusion-activator protein by its cognate kinase results in the transcription of the luciferase gene from the pFR-Luc reporter-plasmid. The intensity of the activation-induced intracellular signal can be measured by carrying out a standard luciferase assay (Fig. 1). To control for transfection efficiency, thymocytes were cotransfected with a plasmid encoding β-galactosidase, which is under the transcriptional control of the cytomegalovirus (CMV) promoter/enhancer. The β-galactosidase activity is used to index the luciferase signal obtained from each sample, as assays for luciferase and β-galactosidase activity can be performed within the same sample tube (see Materials and Methods). This is accomplished using a novel detection system, in which both enzymatic reactions are measured in the form of light emission; that is luciferase on the catalytic breakdown of the substrate luciferin, and β-galactosidase on catalysis of the substrate Galacton-Plus.

### Detection of Signaling Pathways in Stimulated EL4 Cells

Our first aim was to demonstrate that the reporter-plasmids were responsive to known inducers of MAPK or CREB-mediated pathways. Initially, we used electroporation to transfect MAPK-signaling reporter-plasmids into EL4 cells. Our results show that cells transfected with the fusion-activator plasmid, pFA-Elk and the luciferase reporter-plasmid, pFR-Luc and then treated with phorbol ester, phorbol-12-myristate-13-acetate (PMA) and the calcium ionophore, ionomycin show an induction (>200 fold) of luciferase activity, as compared to control untreated cells (Fig. 2a). These results indicated that activation of the MAPK-signaling cascade can be detected following stimulation with PMA/ionomycin. As a positive control, cells co-transfected with a plasmid containing a constitutively active MEK1, pFC-MEK1 showed maximal luciferase activity in the presence or absence of exogenous stimulation (Fig. 2a). Moreover, we have previously demonstrated that the transfection of thymocyte suspensions with pFA-Elk and pFR-Luc, and subsequent stimulation of these cells with PMA/ionomycin led to a similar activation of luciferase activity (Michie and Zúñiga-Pflücker, 2000).

In a similar fashion, we transfected PKA-signaling reporter-plasmids into EL4 cells. Our results show that EL4 cells transfected with pFA2-CREB- and pFR-Luc-plasmids elicited a greater than ten fold stimulation of luciferase activity upon addition of the adenylyl cyclase activator, forskolin, as compared to control untreated cells (Fig. 2b). As a positive control, cells co-transfected with a plasmid containing a constitutively active PKA, pFC-PKA showed a maximal

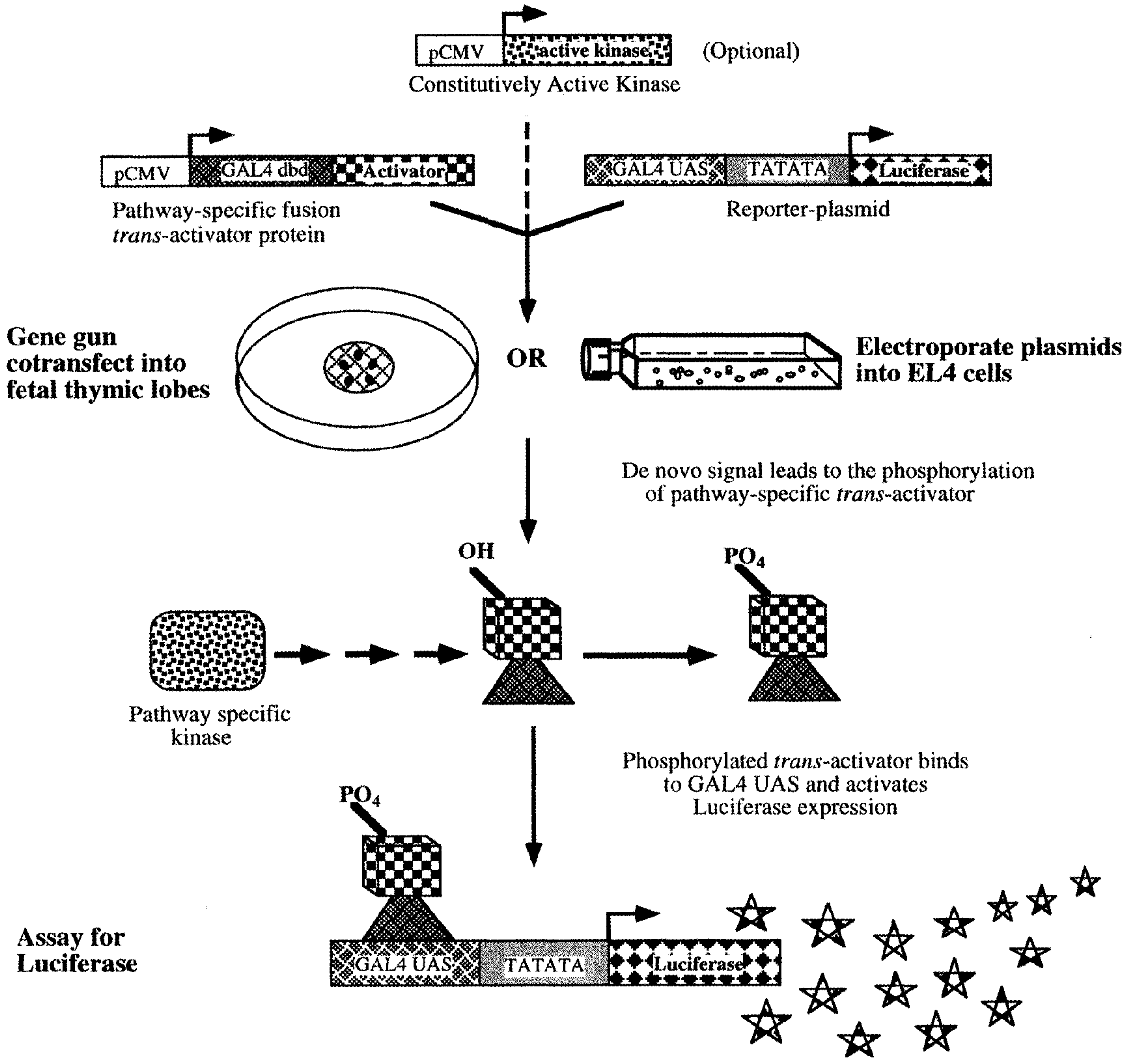

FIGURE 1 *In vivo* signal transduction pathway reporting systems. This experimental system takes advantage of three plasmids: the first plasmid encodes for a fusion protein containing an activation domain derived from the specific substrate recognition site for each kinase (eg., pFA-Elk for MAPK and pFA2-CREB for PKA) and a DNA binding protein domain derived from the yeast DNA transcriptional activator GAL4; the second plasmid encodes for the luciferase gene controlled by five repeats of GAL4 binding element followed by a basic transcriptional promoter (TATATA; pFR-Luc); the third plasmid is the gene of interest or a positive control encoding for constitutively active versions of each of the kinases to be tested, which are under the transcriptional control of strong enhancer/promoter elements (pFC-MEK1 and pFC-PKA). When the fusion-activator plasmid luciferase-reporter plasmid are cotransfected into mammalian cells, phosphorylation of the fusion-activator protein by its cognate kinase leads to the transcriptional activation luciferase gene from the reporter plasmid. The intensity of signal can be measured by carrying out a standard luciferase assay luciferase activity with or without the addition of forskolin (> 40 fold; Fig. 2b).

To demonstrate that these plasmids provided a sensitive readout for biological events, such as anti-

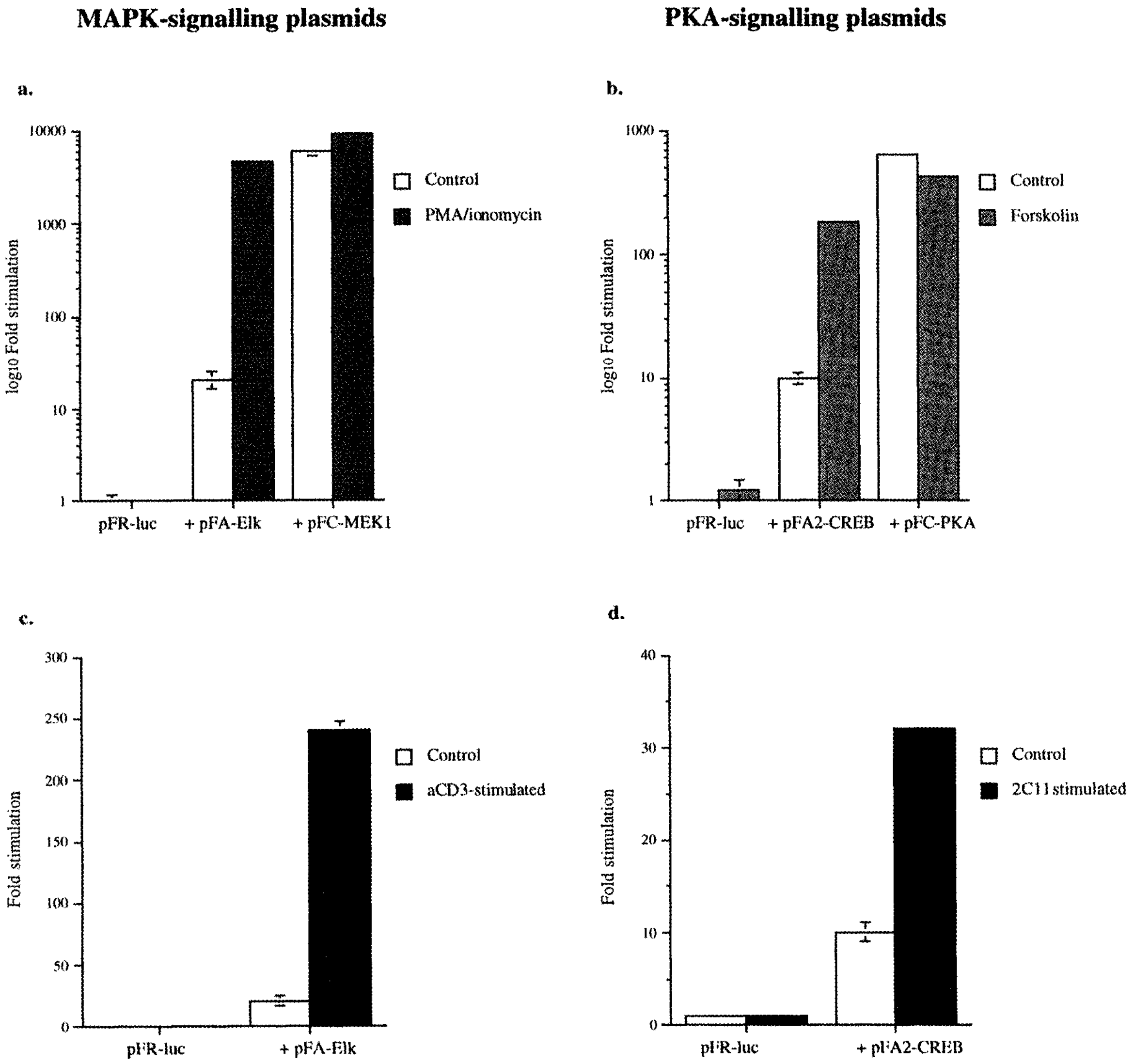

FIGURE 2 Activation of specific signaling cascades upon stimulation of transfected EL4 cells with pharmacologic agents or anti-CD3 monoclonal antibody (145–2C11). EL4 cells were transfected with reporter-plasmids as indicated. MAPK-signaling plasmid transfections are shown in (a) and (c), and PKA-signalling plasmid transfections are shown in (b) and (d). The transfected cells were incubated overnight at 37°C and then stimulated for 6 hr with: (a) PMA/ionomycin (10 ng/ml); (b) forskolin (20 μg/ml); or (c) and (d) plate-bound anti-CD3. Cells were lysed and the lysates were assayed for luciferase and β-galactosidase activity. In each of the conditions, stimulation of the cells transfected with fusion-activator plasmids, pFA-Elk or pFA2-CREB, and the luciferase reporter plasmid, pFR-Luc, leads to an induction of luciferase activity, compared to unstimulated cells. Positive control cells transfected with (a) an active MEK1-plasmid, pFC-MEK1, or (b) an active PKA-plasmid, pFC-PKA, both show maximal luciferase activity with or without stimulation, whereas only background luciferase activity is detected in cells transfected with pFR-Luc alone body-mediated engagement of the TCR/CD3 complex, we transfected EL4 cells with the luciferase reporter-plasmid (pFR-Luc) and either of the fusion-activator plasmids, pFA-Elk or pFA2-CREB, to readout the activation of MAPK- or CREB-mediated pathways, respectively. Our results indicate that engagement of the TCR/CD3 complex, with plate bound anti-CD3-mAb (145–2C11), led to the activation of both MAPK- and CREB-mediated signaling cascades (Figs. 2c and d). Indeed, aggregation of the TCR/CD3 complex in pFA-Elk-transfected cells showed an induction of luciferase activity (>ten fold), compared to unstimulated cells (Fig. 2c), while nearly a four fold stimulation in luciferase activity was observed in pFA2-CREB-transfected cell (Fig. 2d).

### Gene Gun-Mediated Transfection of FTOCs

As previously stated, our aim is to employ the reporter-plasmids to obtain a sensitive readout of signaling events that occur during thymocyte maturation and differentiation. Thus, experiments were carried out using fetal thymic lobes from day 14 timed-pregnant CD1 mice as targets for gene gun-mediated plasmid transfection with a Helios Gene Gun (see Materials and Methods). Our previous experiments have shown that accelerated DNA/gold particles can transfect thymocytes to a depth of six to eight cell layers in the thymus (Zúñiga-Pflücker et al., 1993). Transfection of FTOCs with a plasmid encoding for green fluorescent protein (GFP) bound to gold particles allowed us to determine the transfection efficiency of thymocytes within fetal thymic lobes. Flow cytometric analysis of thymic suspensions obtained from gene gun-transfected FTOCs showed a transfection efficiency of 0.8–3% GFP$^+$ thymocytes. The proportion of thymocytes transfected was determined by staining cell suspensions with a leukocyte-specific marker (CD45) and analyzing for GFP$^+$ cells within the CD45$^+$ population (Fig. 3a). This analysis demonstrates that the GFP$^+$ cells represent developing thymocytes rather than thymic stromal elements, such as the epithelial cells encasing the thymus or cortical epithelial cells within the fetal thymus. Figure 3a shows GFP expression is evident in 1.2% of the CD45$^+$ leukocyte population.

To demonstrate that FTOC transfection by DNA/gold particle bombardment, when combined with the reporter-plasmids provides a reliable and sensitive assay system, we transfected thymic lobes with plasmids-encoding luciferase and β-galactosidase, under the transcriptional control of CMV promoter/enhancer elements. Eighteen hours after transfection, a 35-fold elevation in luciferase activity was observed, as compared to mock-transfected lobes (Fig. 3b). Additionally, β-galactosidase activity was elevated six fold over mock-transfected controls (Fig. 3b). It is apparent from these results that luciferase activity provides a more sensitive readout system for recording the activation of signaling pathways, while β-galactosidase activity exhibits a robust signal, appropriate for indexing purposes.

### Developmental Progression of Transfected Thymocytes Within FTOCs

To further examine the impact of gene gun-transfection on the ability of the thymus to support T lymphopoiesis, and the preservation of the thymic microenvironment, we investigated the longterm developmental potential of transfected thymocytes. Therefore, we transfected recombinase activating gene-2-deficient (RAG-2$^{-/-}$) mouse fetal thymic lobes with a plasmid encoding a constitutively active form of the GTPase Ras, (p21$^{ras(V12)}$) (Downward et al., 1988; Swat et al., 1996). Differentiation of thymocytes from RAG-2$^{-/-}$ mice is halted at the CD117$^-$ CD25$^+$ stage, prior to the generation of DP cells, due to the cells inability to initiate rearrangement of their TCR-β locus (Shinkai et al., 1992). It has been previously shown that the introduction of a transgene encoding an active Ras into RAG$^{-/-}$ mice induces cellular proliferation and differentiation of CD4$^-$ CD8$^-$ DN thymocytes to the CD4$^+$ CD8$^+$ DP stage of development, indicating that Ras plays an essential role during β-selection (Swat et al., 1996).

To demonstrate that transfection of thymocytes in FTOC does not interfere with the normal thymocyte proliferation and developmental progression, consistent with a transition through β-selection, RAG-2$^{-/-}$ FTOCs were gene gun-transfected with plasmid encoding a constitutively active Ras. FTOCs were incubated for 9 days and then analyzed by flow cytometry. In comparison to the mock-transfected (control) FTOCs (Fig. 4a), CD25 expression was downregulated in Ras-transfected FTOCs, with a con-

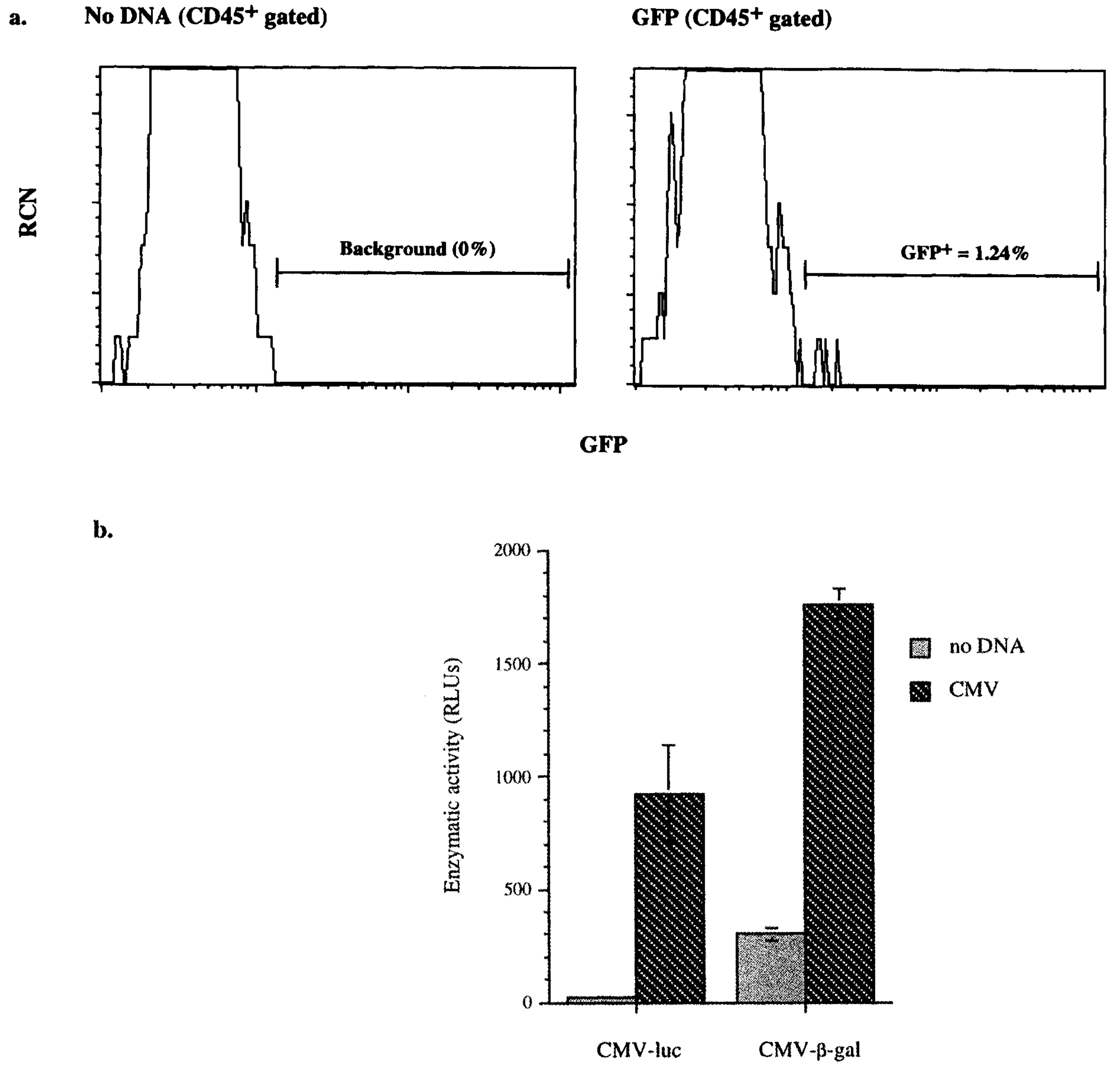

FIGURE 3 Transfection of fetal thymic lobes with: (a) Green Fluorescent Protein (GFP) or (b) plasmids encoding luciferase and β-galactosidase. (a) Intact fetal thymic lobes removed from time-pregnant CD1 mice (day 13 of gestation) were used as targets for DNA-covered gold particles. After 6 hr in culture on nucleopore filters, intact lobes were briefly removed from their gel foam support and subjected to bombardment by accelerated DNA/gold particles (200 psi) using a Helios Gene Gun (Bio-Rad Laboratories). In this experiment, intact lobes were transfected with plasmid DNA encoding GFP (DLR = 1.5 μg). After 18 hr incubation, the lobes were analyzed by flow cytometry for the appearance of GFP within the leukocyte population of cells ($CD45^+$ -gated). Mock transfected cells were bombarded with gold particles alone. Shown is a representative experiment of GFP transfection, showing green fluorescence in 1.24% of the cells within the lymphocyte population. (b) Transfection of fetal thymic lobes with plasmids encoding luciferase and β-galactosidase. Intact fetal thymic lobes removed from timed-pregnant CD1 mice (day 14 of gestation) were used as targets for DNA-covered gold particles, as described. In this experiment, intact lobes were transfected with plasmid DNA-encoding CMV-luciferase and CMV-β-galactosidase (DLR = 250 ng). After 18 hr incubation, the cells were lysed and the resulting lysates were assayed for luciferase and β-galactosidase activity. The results indicate that there is a 35 fold elevation in luciferase activity over mock transfected background and a six fold elevation in β-galactosidase activity over background levels. The data shown is an average of seven independent experiments comitant upregulation of both CD8 and CD4 molecules, indicating that differentiation to the $CD4^+$ $CD8^+$ DP stage of development has occurred (Fig. 4b). Moreover, an increase in thymic cellularity was evident in Ras-transfected FTOCs compared with mock-transfected (control) FTOCs ($2.4 \times 10^4$ vs. $0.56 \times 10^4$ cells/lobe, respectively). Therefore, these results suggest that gene gun-transfection does not impair the thymic microenvironment and leaves the thymic stromal cells functionally intact. Indeed, this finding mirrors the developmental progression observed in transgenic mice (Swat et al., 1996), thus supporting the notion that the thymic microenvironment and the signals generated within transfected thymocytes are the same as those produced *in vivo*.

### Detection of Activated Signaling Pathways in FTOCs

In order to define the conditions required for the optimal readout of MAPK- and PKA- signaling pathways, thymic lobes were transfected with increasing amounts of pFA-Elk or pFA2-CREB plasmids, while maintaining constant levels of the reporter- and indexing-plasmids (Figs. 5 and 6). Induction of luciferase activity was apparent in FTOCs transfected with reporter-plasmids (pFA-Elk, pFR-Luc, and β-galactosidase) and then stimulated with PMA/ionomycin (Fig. 5). Although an increase in the amount of pFA-Elk caused a slight elevation in background luciferase activity, the addition of PMA/ionomycin to transfected FTOCs led to a clear induction of luciferase activity in each sample. Maximum stimulation of luciferase activity, at nearly three fold stimulation over background, was seen in FTOCs transfected with 250 ng of pFA-Elk (Fig. 5). At this amount, the ratio between fusion-activator- and reporter-plasmids (pFA-Elk: pFR-Luc) is 1:1. Thus, these two plasmids should be used at this ratio to gain optimal readout of the MAPK signalling cascade in fetal thymic lobes.

Similarly, we have defined the optimal conditions for detecting CRER-mediated signals in thymocytes within thymic lobes. FTOCs were transfected by DNA/gold bombardment with reporter-plasmids (pFA2-CREB, pFR-Luc and β-galactosidase) and then treated with forskolin. The lobes were transfected with increasing amounts of pFA2-CREB while keeping the concentration of the other plasmids constant (Fig. 6). An elevation in background luciferase activity was not evident on increasing the concentration of pFA2-CREB plasmid. However, the addition of forskolin led to a significant stimulation of luciferase activity in every sample, with the maximum induction (> four fold stimulation) detected at 75 ng of pFA2-CREB (Fig. 6). This result indicates that the optimal ratio of pFA2-CREB: pFR-Luc for attaining maximal readout of PKA activity in fetal thymic lobes is 0.3:1. Thus, these two plasmids should be used at this ratio to gain optimal readout of the PKA signalling cascade in FTOCs.

Our findings demonstrate that despite the low transfection efficiency of FTOCs by gene gun-bombardment, the sensitivity of the readout system clearly allows for the study of biochemical signaling cascades in real-time and in a relevant biological setting. The use of this novel and powerful model system provides an important tool for the further elucidation of signaling pathways regulating key checkpoints during T cell development in the thymus.

### DISCUSSION

The method described in this paper relies on the combination of two experimental approaches: (1) a reporter-plasmid system enabling the detection of *de novo* intracellular signaling pathways *in vivo* and, (2) a method for the transfection of thymocytes within an intact thymic microenvironment (Michie and Zúñiga-Pflücker, 2000; Zúñiga-Pflücker et al., 1993). To this end, we have developed the use of reporter-plasmids together with a DNA/particle bombardment delivery system to transfect thymocytes in FTOC, permitting the study of biochemical signaling events in real time and within a relevant biological setting. One application of this approach involved the characterization of MAPK- and CREB-mediated intracellular signalling pathways within the thymus.

There are many advantages of this transfection system compared with other methods available: (1) the

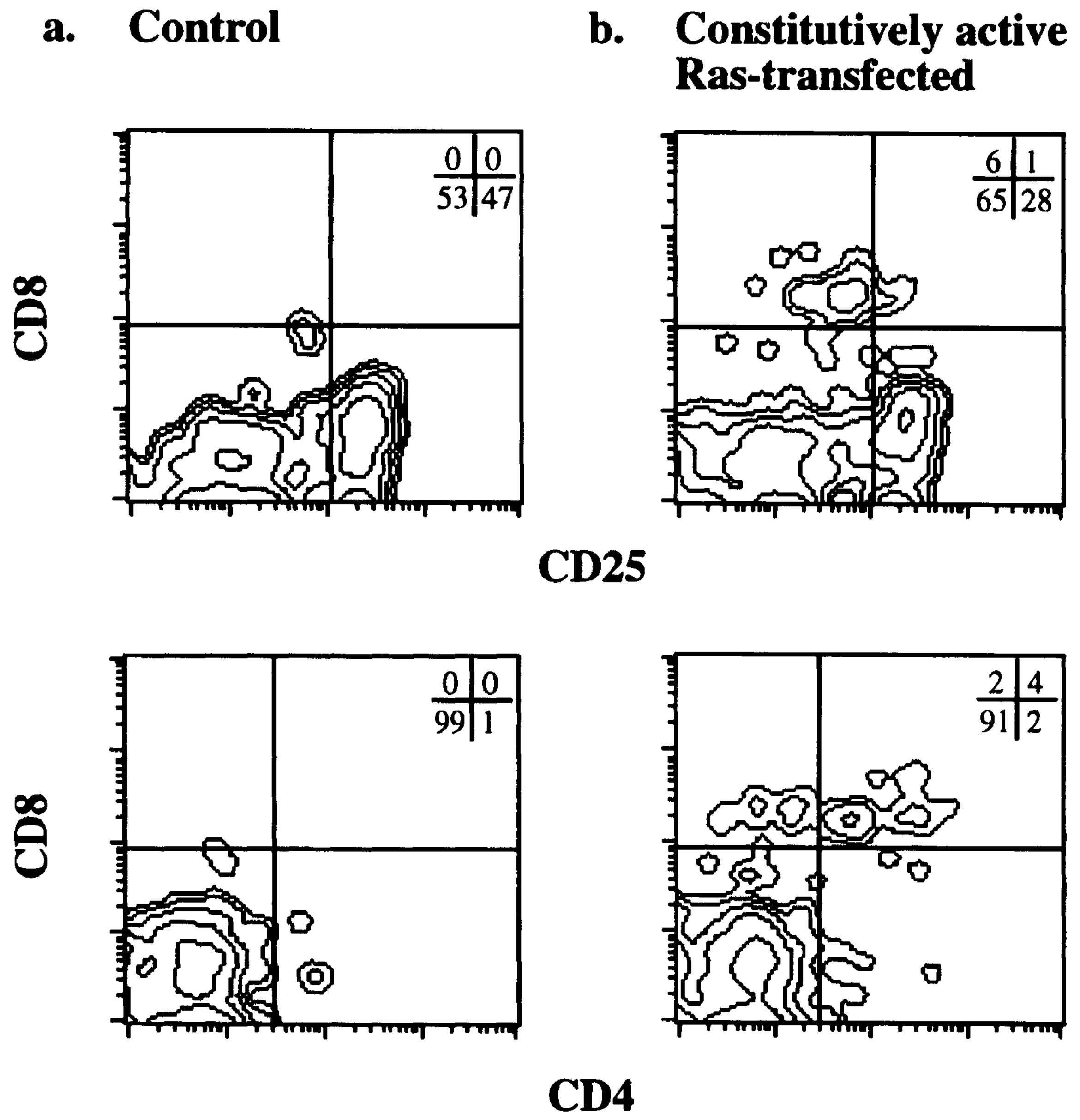

FIGURE 4 Generation of CD4$^+$ CD8$^+$ double positive cells in RAG-2$^{-/-}$ lobes transfected with constitutively active Ras. Intact fetal thymic lobes removed from timed pregnant RAG-2$^{-/-}$ mice (day 14 of gestation) were used as targets for DNA-covered gold particles, as described in the legend to Fig. 3. In this experiment, fetal thymuses were gene gun-transfected with either (a) no DNA (control) or (b) constitutively active Ras (Ha-Ras (V12); DLR = 750 ng), and then cultured for nine days in FTOC. After this time, a single cell suspension of the fetal thymic lobes was prepared and thymocytes were analyzed for surface expression of CD25, CD8 and CD4, by flow cytometry specific signaling cascades can be read out *in vivo* and within the thymic environment; (2) multiple plasmids can be transfected simultaneously, enabling the study of direct or indirect interactions between proteins; (3)

the transfection process is rapid and without apparent alteration of the thymic microenvironment; (4) the entire procedure requires less than 48 hours to complete; (5) this method offers a cost-effective means to study signaling components during T cell development; (6) the amount of tissue required is small (4 to 6 fetal thymic lobes/ transfection); (7) the amount of DNA required is small (25–250 ng); and (8) despite the transient nature of the transfection, it may be possible to examine long term developmental effects following the introduction of a particular gene at a specific stage of development.

The key signaling components required for lymphocyte development have been largely determined by phenotypic and molecular characterization of mice with targeted gene deficiencies, and by the introduction of dominant negative or constitutively active kinases into developmentally blocked mice (von Boehmer et al., 1999). While these studies have provided invaluable information, they are time consuming, costly, and do not provide direct and detailed evidence for signaling mechanisms that emanate from the gene product of interest. Our gene-gun transfection technique, coupled with the reporter-plasmid system, should allow for the detection of signaling cascades generated from the activation of upstream kinases. To this end, it is important to point out that our present findings validate this approach by showing that transfection of RAG-deficient DN thymocytes with a constitutively active Ras induced the developmental progression to the DP stage, and thus experimentally bypassed pre-TCR-mediated signals.

It is well known that removal of thymocytes from their microenvironment severely interferes with their normal developmental processes and leads to the induction of apoptosis (Fisher et al., 1996). Indeed, it has been demonstrated that perturbation of thymic stromal interactions with developing T cells leads to a spontaneous upregulation of TCR-$\alpha\beta$ complex on the surface of the cells together with dephosphorylation of the CD3$\zeta$ chain (Nakayama et al., 1990). Moreover, thymocytes cultured in suspension or within intact lobes respond differently to stimulation with PMA, mitogen Concanavalin A, or anti-CD3 (Anderson and Jenkinson, 1998; Fisher et al., 1996). These and other studies indicate that the interactions within the intact thymic environment play an essential role in determining the biological outcome to specific stimuli. Therefore, the method described here is of central importance, as it provides an important tool to the study the regulation of T cell development by enabling researchers to define signaling mechanisms within the cell's natural environment.

Although gene gun-mediated transfection of FTOCs allows thymocytes to respond to their normal environment, other means of *in vitro* gene transfer also confer this ability. In particular, retroviral-mediated infection of cells has proven to be a viable means with which to study specific gene function during thymocyte differentiation (Crompton et al., 1996; Pallard et al., 1999; Sugawara et al., 1998). However, one main benefit of the gene-gun system is the ability to transfect multiple plasmids into thymocytes, which can be read out in short or long term assays. Thus, this system permits the expression of specific genes during T cell development, together with the use of a separate reporter-plasmid allowing for the readout of *de novo* signaling events.

### MATERIALS AND METHODS

### Animals

Timed-pregnant CD1 mice were obtained from the Charles River Laboratories (St. Constant, QC Canada). Recombinase activating gene-2-deficient (RAG-2$^{-/-}$) mice (Shinkai et al., 1992) were bred and maintained in our animal facility. Timed-pregnant RAG-2$^{-/-}$mice were generated and the fetuses were extracted at day 14 of pregnancy.

### Electroporation of EL4 Cells

All plasmid DNA used for transfection was purified by anion-exchange chromatography using Qiagen columns (Valencia, CA). PathDetect reporter-plasmids were purchased from Stratagene (La Jolla, CA). EL4 cells were washed and placed in RPMI-1640

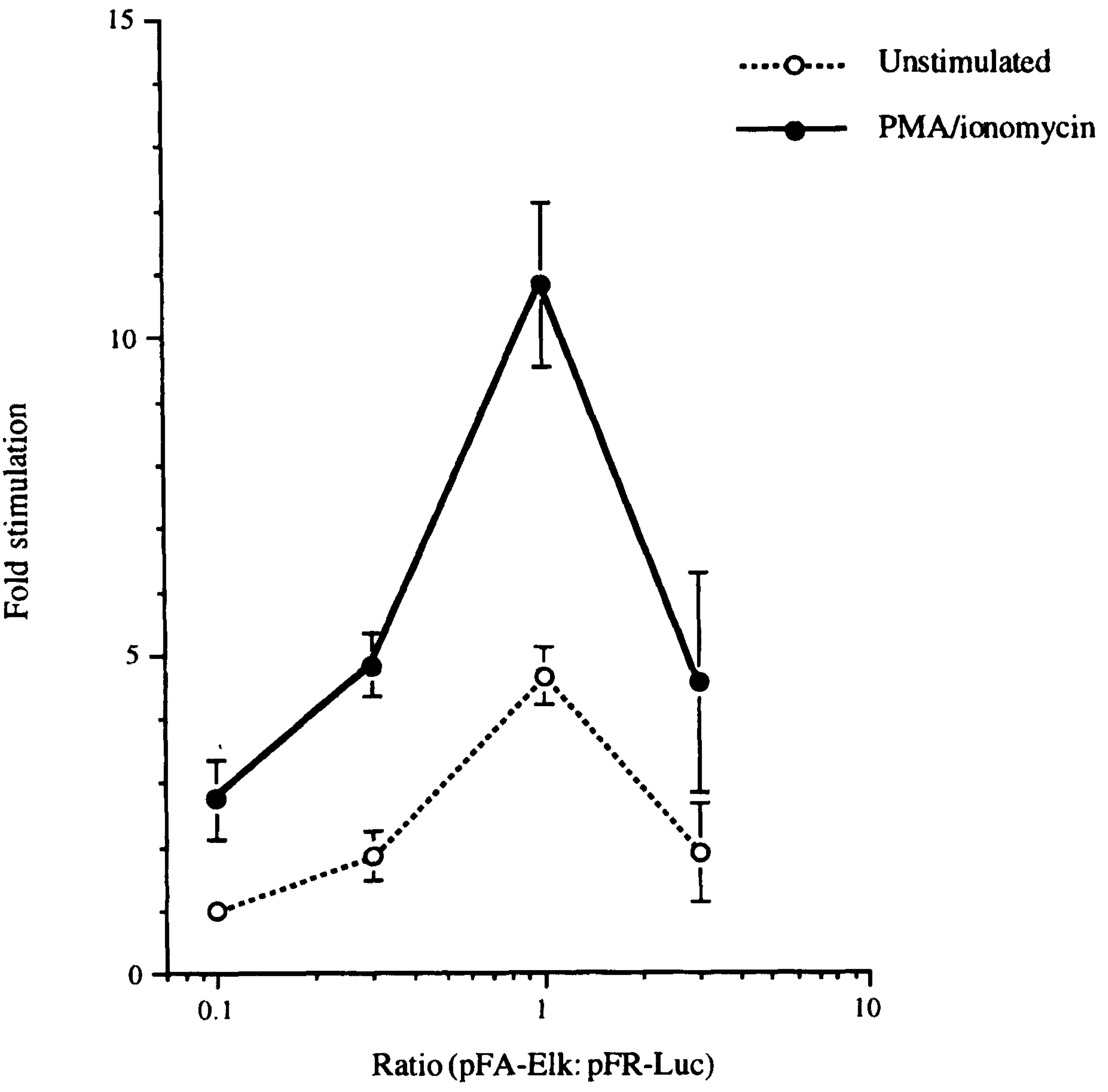

FIGURE 5 Activation of MAPK signalling cascade on stimulation of fetal thymic lobes with phorbol ester, phorbol-12-myristate-13-acetate (PMA) and the calcium ionophore, ionomycin. Intact fetal thymic lobes removed from timed-pregnant CD1 mice (day 14 of gestation), were used as targets for DNA-covered gold particles, as described in the legend of fig. 3. Fetal thymuses were gene gun-transfected with pFR-Luc (DLR = 250 ng), pFA-Elk (DLR = 25–750 ng), and CMV-β-gal (DLR = 250 ng), and then cultured for 12 to 18 hr prior to addition of PMA/ionomycin (10 ng/ml of each) for a further 6 to 8 hr. Cells were lysed and the lysates were assayed for luciferase and β-galactosidase activity. The optimal concentration of pFA-Elk in fetal thymic lobes is 250 ng/shot (DLR = 250 ng), as almost a three fold stimulation is observed in luciferase activity and thus MAPK activity. The results shown are an average of four separate experiments with each condition carried out in triplicate medium supplemented with 20% FCS. Cells were transfected by electroporation using a BTX (San Diego, CA) Electro Cell Manipulator 600 apparatus with each sample containing 1–3 × $10^6$ cells per cuvette (4-mm gap) in 250 μl medium. Samples were incubated on ice for 10 min with 21–50 μg plasmid DNA (as indicated in figure legends) and then electroporated at 250 V, 1200 μF, 186 Ω with a ~60 ms time constant. After electroporation, samples were incubated on ice for 10 min, and then cultured for 18 to 24 hr in DMEM-high glucose media (Gibco-BRL, Gaithersburg, MD) supplemented with 10% FCS, 2 mM glutamine, 10 U/ml penicillin, 100 μg/ml streptomycin, 100 pg/ml gentamicin, 110 μg/ml sodium pyruvate, 50 μM 2-mercaptoethanol, and 10 mM Hepes, pH 7.4 (EL4 medium). Phorbol-12-myristate-13-acetate (PMA), ionomycin, or forskolin (Sigma, St. Louis, MO) were added to the cells as described in the figure legends.

### Preparation of DNA/Gold Suspension

The DNA/gold bound cartridges were prepared as described by the manufacturer's protocol (Bio-Rad Laboratories, Hercules, CA). The amount of microcarriers (gold powder, spherical (1.5 to 3.0 microns; Aldrich Chemical Co., Milwaukee, WI)) to be delivered to the target (fetal thymic lobes) is referred to as the Microcarrier Loading Quantity (MLQ). Our protocol is optimized for an MLQ of 1 μg of gold. The amount of DNA loaded per microgram of microcarriers is referred to as the DNA Loading Ratio (DLR). In this protocol, the DLR for total plasmid DNA is 0.65 : 1.4. It should be noted that, as the MLQ = 1 μg, the DLR for each plasmid (detailed in the figure legends) is equivalent to the amount of DNA delivered to the fetal thymic lobes in each transfection. To prepare the DNA/gold suspension, 100 μl of 0.05 M spermidine was added to 40 μg of gold particles. This solution was vortexed and sonicated for 3 to 5 seconds to remove gold clumps. To the gold/spermidine mixture, DNA (1 to 30 μg of each plasmid) was added to a final volume not exceeding 100 μl, and vortexed. While vortexing, the DNA was precipitated onto gold particles by adding 100 μl 1 M $CaCl_2$ dropwise. This was left to stand for 10 min at room temperature (RT), then the gold/DNA bound particles were pelleted. The supernatant was removed and the pellet was washed 3 times in anhydrous ethanol (Aldrich Chemical Co.). After the final wash, the pellet was resuspended in 2.4 ml anhydrous ethanol containing 0.1% polyvinylpyrrolidone (PVP; Bio-Rad Laboratories) and kept in an airtight tube. The DNA/gold suspension is ready for cartridge preparation, however it can be stored for up to 2 months at –20°C.

### Preparation of DNA/Gold-Coated Cartridges

In order to generate DNA/gold bound cartridges, a Tubing Prep Station is required (Bio-Rad Laboratories). Immediately prior to preparing cartridges, the Gold-Coat tubing (Bio-Rad Laboratories) was dried by purging with nitrogen for 15 min. Seventy-five centimeters of dried tubing was then placed in the Tubing Prep Station. The DNA/gold particle suspension was vortexed, then drawn into a syringe and injected into the dried tubing. The microcarriers were allowed to settle for 3 to 5 min then the ethanol/PVP solution was slowly drawn back into the syringe, leaving the DNA-bound gold in the tubing. The syringe was removed and the Gold-Coat tubing was immediately turned 180° to allow the gold to coat the inside of the tubing. After 10 seconds, the tubing is rotated for 3 to 5 min, thus coating the tube with gold/DNA particles. Following the tube coating with the DNA/gold, the tubing was dried by passing nitrogen for 3 min. The DNA/gold-coated tube was removed from the Tubing Prep Station, and cut into 1.25, cm cartridges using a Tubing Cutter (Bio-Rad Laboratories). The DNA/gold slurry (2.4 ml) generates 40 cartridges with the desired MLQ. The prepared cartridges can be stored at 4°C for up to 8 months.

### Transfection of Fetal Thymuses

Fetal thymuses isolated from gestational day 13–14 time-pregnant mice were placed on prewetted Nucleopore filters (13 mm, 0.8 μm pore size, polycarbonate) (Corning, NY), 4 to 6 lobes per filter in FTOC media (EL4 media supplemented with a further 5% FCS). The filters were placed on top of media-soaked Gelfoam (Upjohn Canada, Don Mills, ON) rafts.

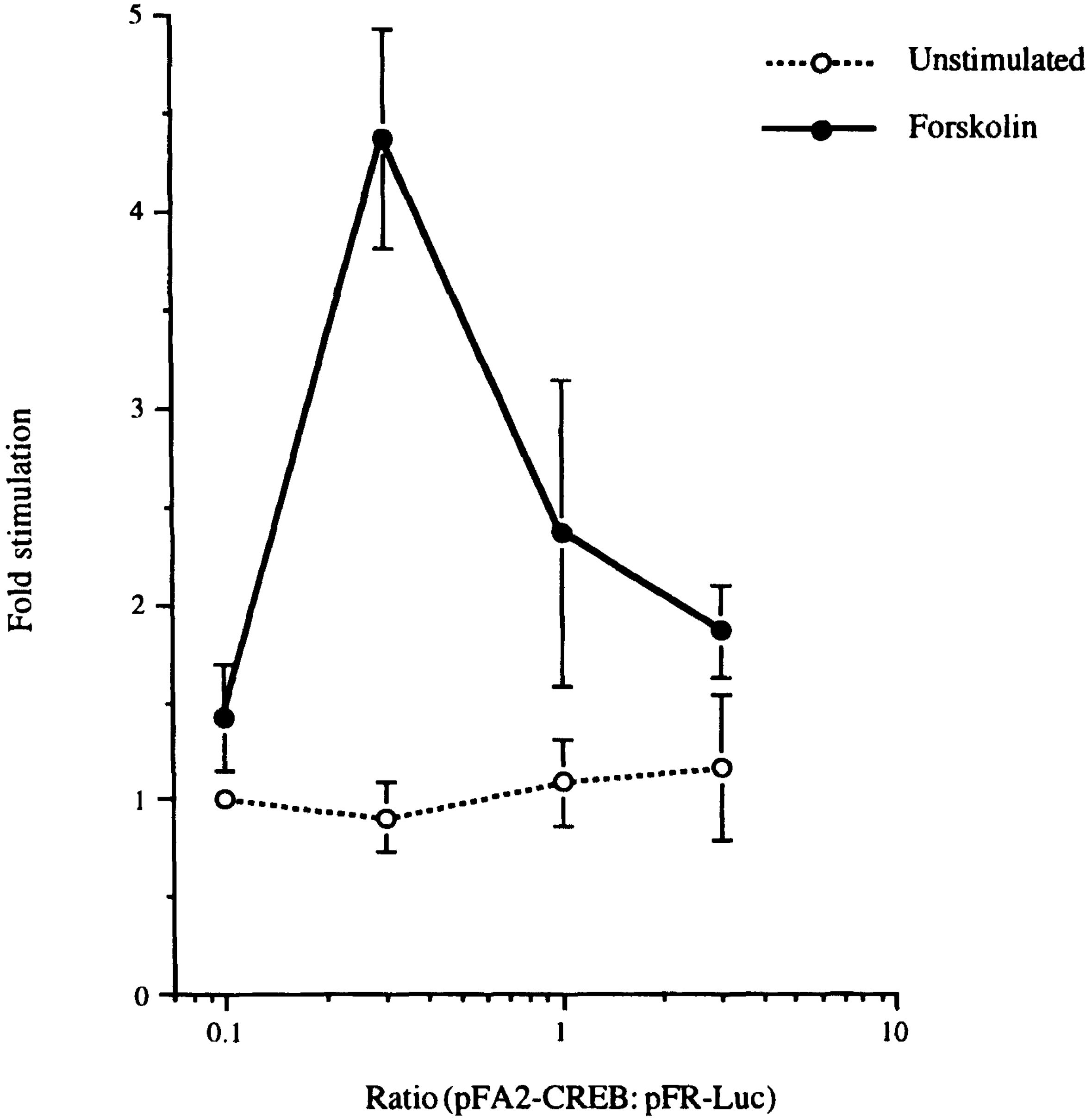

FIGURE 6 Activation of PKA signaling cascade on stimulation of fetal thymic lobes with the adenylyl cyclase activator, forskolin. Intact fetal thymic lobes removed from timed-pregnant CD1 mice (day 14 of gestation) were used as targets for DNA-covered gold particles, as described in the legend of Fig. 3. Fetal thymuses were gene gun-transfected with pFR-Luc (DLR = 250 ng), pFA-CREB (DLR = 25–750 ng), and CMV-β-gal (DLR = 250 ng), and then cultured for 12 to 18 hr prior to addition of forskolin (20 μg/ml) for a further 6 to 8 hr. Cells were lysed and the lysates were assayed for luciferase and β-galactosidase activity. The optimal concentration of pFA2-CREB-activator in fetal thymic lobes is 75 ng/shot (DLR = 75 ng), as > four fold stimulation is seen in luciferase activity and thus PKA activity. The results shown are an average of six separate experiments with each condition carried out in triplicate

After 6 hr, the filters carrying the fetal thymic lobes were transferred onto a petri dish (6 mm), and immediately transfected by gene gun-bombardment with a Helios Gene gun set at 200 psi (Bio-Rad Laboratories), using one cartridge per set of lobes. The filters carrying the transfected fetal lobes were then placed back onto the Gelfoam rafts and incubated at 37°C for 8–24 hr in a humidified incubator with 5% $CO_2$. Alternatively, the transfected lobes were incubated for 4 to 6 hr prior to the addition of pharmacological reagents as stated in the figure legends. Thereafter, the lobes were incubated for an additional 12 to 18 hr prior to analysis. For long-term developmental progression analysis, transfected fetal thymic lobes were incubated for 9 days, changing FTOC media once during the incubation.

### Luciferase and β-Galactosidase Assay

A single-cell suspension was prepared in phosphate-buffered saline (PBS; Gibco-BRL) from transfected thymic lobes by crushing the lobes through a prewetted 70 μm nylon mesh filter (BioDesign, Carmel, NY). The cells were then assayed for luciferase and β-galactosidase activities using the Dual-Light reporter gene assay system (Tropix, Perkin Elmer-Applied Biosystems, Norwalk, CT). Briefly, thymocytes were lysed in 25 μl Lysis Buffer (40 mM Tricine, pH 7.8, 50 mM NaCl, 2 mM EDTA, 1 mM $MgSO_4$, 5 mM dithiothrietol (DTT), 1% Triton X100) for 15 min at RT, and then centrifuged (13,000 g) for 5 min to remove cell debris. The supernatant was combined with an equal volume of Luciferase Reaction Buffer (30 mM Tricine, pH 7.8, 3 mM ATP, 15 mM $MgSO_4$, 1 mM Coenzyme A, 10 mM DTT), and after addition of 1 mM luciferin (100 μl) the sample was immediately assayed for luciferase activity, with a Lumat LB 9507 Luminometer (Fischer Scientific Canada, Unionville, ON), with light emission read out for 10 seconds. To assay for β-galactosidase activity, Galacton-Plus (substrate for β-galactosidase; Tropix) was added to each tube after the luciferase assay was completed. The tubes were incubated at RT for 30 to 60 min, then Accelerator-II (100 μl; Tropix) was added to each tube and the samples were immediately assayed for β-galactosidase activity, measured as light emission with a Lumat LB 9507 Luminometer, with light emission read out for 10 seconds. Results shown represent the averages of assays carried out in triplicate.

### Flow Cytometric Analysis

Transfected fetal thymic lobes were incubated at 37°C for the desired time (as described in figure legends), then single-cell suspensions were prepared. The cells were incubated with anti-mouse antibodies in FACS buffer (Hank's balanced salt solution (without phenol red) containing 1% BSA and 0.05% $NaN_3$) for 30 min on ice. The antibodies used were as follows: FITC-conjugated CD25 (7D4); R-PE-conjugated CD8α (53–6.7); APC-conjugated CD4 (RM4–5), or biotinylated CD45 (30F11.1) purchased from Pharmingen (San Diego, CA). The stained thymocytes were then washed twice in FACS buffer and, where biotinylated antibodies were used, incubated for a further 20 min on ice in the presence of Strepta-vidin-APC (Pharmingen). Stained cells were washed twice in FACS buffer and analyzed with a FACSCaliber flow cytometer using CELLQuest software (Becton-Dickinson, Mountain View, CA); data was live-gated by size and lack of propidium iodide uptake.

### *Acknowledgements*

We would like to thank Dr. Pamela Ohashi for the plasmid encoding constitutively active Ras.

A. M. Michie is supported by a postdoctoral fellowship from the Cancer Research Institute. J. C. Zúñiga-Pflücker is supported by a scholarship from the Medical Research Council of Canada. This work is funded by a grant from the Leukemia Research Fund of Canada.

### *References*

Anderson, G., and Jenkinson, E.J. (1998). Use of Explant Technology in the Study of In Vitro Immune Responses. J. Immunolog. Methods *216*; 155–163.

Anderson, G., Moore, N.C., Owen, J.J., and Jenkinson, E.J. (1996). Cellular interactions in thymocyte development. Annu. Rev. Immunol. *14*; 73–99.

Clements, J.L., Yang, B., Ross-Barta; S. E., Eliason, R.F., Williamson, R.A., and Koretzky, G.A. (1998). Requirement for the Leukocyte-Specific Adapter Protein SLP-76 for normal T cell development. Science *281*: 416-419.

Crompton, T., Gilmour, K.C., and Owen, M.J. (1996). The MAP kinase pathway controls differentiation from double-negative to double positive thymocyte. Cell *86*: 243–251.

Downward, J., de Gunzburg, J., Riehl, R., and Weinberg, R.A. (1988). p21ras-induced responsiveness of phosphatidylinositol turnover to bradykinin is a receptor number effect. Proc. Natl. Acad. Sci. USA *85*: 5774–5778.

Fisher, G.H., Lenardo, M.J., and Zúñiga-Pflücker, J.C. (1996). Synergy between T cell receptor and Fas (CD95/APO-1) signalling in mouse thymocyte death. Cell. Immunol. *169*; 99–106.

Fowlkes, B.J., and Schweighoffer, E. (1995). Positive selection of T cells. Curr. Opin. Immunol. *7*, 188–195.

Galandrini, R., Henning, S.W., and Cantrell, D.A. (1997). Different Functions of the GTPase Rho in Prothymocytes and Late Pre-T cells. Immunity *7*; 163–174.

Godfrey, D.I., Kennedy, J., Suda, T., and Zlotnik, A. (1993). A developmental pathway involving four phenotypically and functionally distinct subsets of $CD3^-CD4^-CD8^-$ triple negative adult mouse thymocytes defined by CD44 and CD25 expression. J. Immunol. *150*; 4244–4252.

Godfrey, D.I., and Zlotnik, A. (1993). Control points in early T-cell development. Immunol. Today *14*; 547–553.

Hoffman, E.S., Passoni, L., Crompton, T., Leu, T.M.J., Schatz, D.G., Koff, A., Owen, M.J., and Hayday, A.C. (1996). Productive T-cell receptor β-chain gene rearrangement: coincident regulation of cell cycle and clonality during development in vivo. Genes and Dev. *10*; 948–962.

Jenkinson, E.J., Franchi, L., Kingston, R., and Owen, J.J.T. (1982). Effects of deoxyguanosine on lymphopoiesis in the developing thymus rudiment in vitro: application in the production of chimeric thymus rudiments. Eur. J. Immunol. *12*, 583.

Kisielow, P., and von Boehmer, H. (1990). Negative and positive selection on immature thymocytes: timing and the role of ligand for α/β T cell receptor. Semm. Immunol. *2*; 35–44.

Levin, S.D., Anderson, S.J., Forbush, K.A., and Perlmutter, R.M. (1993). A dominant-negative transgene defines a role for $p56^{lck}$ in thymopoiesis. EMBO J. *12*; 1671–1680.

Michie, A.M., and Zúñiga-Pflücker, J.C. (2000). Transfection and Transcription of Genes in Developing Thymocytes. In Methods in Molecular Biology: Protocols in T cell Development and Activation, K.P. Kearse, ed. (Totawa, NJ: Humana Press), *134*; 55-62.

Molina, T.J., Kishihara, K., Siderovski, D.P., van Ewijk, W., Navendran, A.W., Timms, E., Wakeham, A., Paige, C.J., Hartmann, K.U., Veillette, A., Davidson, D., and Mak, T.W. (1992). Profound block in thymocyte development in mice lacking $p56^{lck}$. Nature *357*; 161–164.

Nakayama, T., June, C.H., Munitz, T.I., Sheard, M., McCarthy, S.A., Sharrow, S.O., Samelson, L. E., and Singer, A. (1990). Inhibition of T cell receptor expression and function in mature CD4+ CD8+ cells by CD4. Science *249*; 1558.

Negishi, I., Motoyama, N., Nakayama, K., Senju, S., Hatakeyama, S., Zhang, Q., Chan, A.C., and Loh, D.Y. (1995). Essential role for ZAP-70 in both positive and negative selection of thymocytes. Nature *376*; 435–438.

Nickoloff, J.A. (1995). Animal cell electroporation and electrofusion Protocols. In Methods in Molecular Biology, J.A. Nickoloff, ed. (Totowa, NJ: Humana Press) *48*; 115-121.

Pallard C., Stegmann, A P., van Kleffens, T., Smart; F., Venkitaraman, A., and Spits, H. (1999). Distinct roles of the phosphatidylinositol 3-kinase and STAT5 pathways in IL-7-mediated development of human thymocyte precursors. Immunity *10*; 525–535.

Rooke, R., Waltzinger, C., Benoist, C., and Mathi, D. (1997). Targeted complementation of MHC class II deficiency by intrathymic delivery of recombinant adenoviruses. Immunity *7*; 123–134.

Shinkai, Y., Rathbun, G., Lam, K.-P., Oltz, E.M., Stewart, V., Mendelsohn, M., Charron, J., Datta, M., Young, F., Stall, A.M., and Alt, F.W. (1992). RAG-2-deficient mice lack mature lymphocytes owing to inability to initiate V(D)J rearrangement. Cell *68*; 855–867.

Shortman, K., and Wu, L. (1996). Early T lymphocyte progenitors. Ann. Rev. Immunol. *14*; 29–47.

Sugawara, T., Di Bartolo, V., Miyazaki, T., Nakauchi, H., Acuto, O., and Takahama, Y. (1998). An improved retroviral gene transfer technique demonstrates inhibition of CD4-CD8-thymocyte development by kinase-inactive ZAP-70. J. Immunol. *161*; 2888–2894.

Suzuki, H., Zelphati, O., Hildebrand, G., and Leserman, L. (1991). CD4 and CD7 molecules as targets for drug delivery from antibody bearing liposomes. Exp. Cell. Res. *193*; 112–119.

Swan, K.A., Alberola-Ila, J., Gross, J.A., Appleby, M.W., Forbush, K.A., Thomas, J.F., and Perlmutter, R.M. (1995). Involvement of p21ras distinguishes positive and negative selection in thymocytes. EMBO J *14*; 276–285.

Swat, W., Shinkai, Y., Cheng, H.L., Davidson, L., and Alt, F.W. (1996). Activated ras signals differentiation and expansion of CD4+CD8+ thymocytes. Proc. Natl. Acad. Sci. USA *93*; 4683–4687.

von Boehmer, H., Aifantis, I., Feinberg, J., Lechner, O., Saint-Ruf, C., Walter, U., Buer, J., and Azogui, O. (1999). Pleiotropic changes controlled by the pre-T-cell receptor. Curr. Opin. Immunol. *11*; 135–142.

von Boehmer, H., and Fehling, H.J. (1997). Structure and function of the pre-T cell receptor. Annu. Rev. Immunol. *15*; 433–452.

Zhang, W., Sommers, C.L., Burshtyn, D.N., Stebbins, C.C., DeJarnette, J.B., Trible, R.P., Grinberg, A., Tsay, H.C., Jacobs, H.M., Kessler, C.M., Long, E.O., Love, P.E., and Samelson, L. E. (1999). Essential role of LAT in T cell development. Immunity *10*; 323–332.

Zúñiga-Pflücker, J.C., and Lenardo, M.J. (1996). Regulation of thymocyte development from immature progenitors. Curr. Opin. Immunol. *8*; 215–224.

Zúñiga-Pflücker, J.C., Schwartz, H.L., and Lenardo, M.J. (1993). Gene transcription in differentiating immature T cell receptor$^{neg}$ thymocytes resembles antigen-activated mature T cells. J. Exp. Med. *178*; 1139–1149.

